# Stabilized convection in a ternary mixture with two Soret coefficients of opposite sign

**DOI:** 10.1140/epje/s10189-022-00202-5

**Published:** 2022-05-28

**Authors:** Loreto García-Fernández, Henri Bataller, Paul Fruton, Cédric Giraudet, Alberto Vailati, Fabrizio Croccolo

**Affiliations:** 1 E2S UPPA, CNRS, TotalEnergies, LFCR UMR5150, Universite de Pau et des Pays de l’Adour, Anglet, France; 2 Centre National d’Études Spatiales (CNES), 2, Place Maurice Quentin, Paris, France; 3 Department of Structure of Matter, Thermal Physics and Electronics, Faculty of Physics, University Complutense of Madrid, Madrid, Spain; 4 Dipartimento di Fisica “A. Pontremoli”, Università degli Studi di Milano, Milan, Italy

## Abstract

**Abstract:**

We performed ground-based experiments on the sample polystyrene–toluene–cyclohexane in order to complement the experimental activities in microgravity conditions related to the ESA projects DCMIX4 and Giant Fluctuations. After applying a stabilizing thermal gradient by heating from above a layer of the fluid mixture, we studied over many hours the density variations in the bidimensional horizontal field by means of a Shadowgraph optical setup. The resulting images evidence the appearance of convective instability after a diffusive time associated with the binary molecular solvent consisting of toluene and cyclohexane, confirming the negative sign of the Soret coefficient of this mixture. After a larger diffusive time related to mass diffusion of the polystyrene in the binary solvent, convection was suppressed by the increasing stabilizing density gradient originated by the Soret-induced concentration gradient of the polymer. This is compatible with a positive sign of the Soret coefficient of the polymer in the binary solvent.

**Graphic Abstract:**

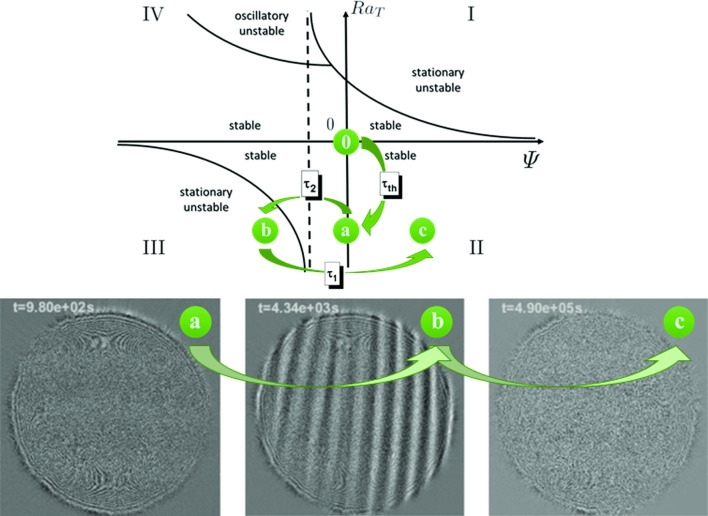

**Supplementary Information:**

The online version contains supplementary material available at 10.1140/epje/s10189-022-00202-5.

## Introduction

It is widely known, even outside the scientific community, that a single component fluid heated from below can undergo convective instability, if the applied temperature gradient is large enough and the dimensionless (thermal) Rayleigh number:

1 $$\begin{aligned} Ra_T=\frac{\beta _T\mathbf {g}\cdot \mathbf {\nabla } T }{\nu \kappa }L^4, \end{aligned}$$

exceeds the critical value $Ra_{T_c}=1707.8$ [1], valid in the case of our geometry. Here, $\beta _T=-\rho ^{-1} (\partial \rho /\partial T)$ is the thermal expansion coefficient of the fluid, $\rho$ the fluid density, *T* the temperature, $\mathbf {g}$ the gravitational acceleration, $\mathbf {\nabla } T$ the temperature gradient, *L* the vertical extension of the sample, $\nu$ the kinematic viscosity and $\kappa$ the thermal diffusivity.

By contrast, a single component fluid is, in general, gravitationally stable if heated from above, as witnessed by the resulting negative sign of the Rayleigh number. A relevant exception is provided by water, which has a negative thermal expansion coefficient below $4^\circ \hbox {C}$ at atmospheric pressure.

In the case of a binary liquid mixture, the situation becomes more complex, because, when the mixture is heated from above, the density gradient induced by thermal expansion points downwards, but the direction and amplitude of the density gradient induced by solutal expansion both depend on the Soret coefficient $S_T$ and, thus, the sign of the solutal Rayleigh number:

2 $$\begin{aligned} Ra_\omega =-\frac{\beta _{\omega } \mathbf {g}\cdot \mathbf {\nabla } \omega }{\nu D}L^4, \end{aligned}$$

where $\beta _\omega =\rho ^{-1}(\partial \rho /\partial \omega )$ is the solutal expansion coefficient, $\mathbf {\nabla } \omega$ the concentration gradient in mass fraction and *D* the Fick diffusion coefficient. The fluid behaviour at the steady state can be then predicted by looking at the separation ratio $\psi$:

3 $$\begin{aligned} \psi =\omega (1-\omega )S_T{\beta _{\omega }}/{\beta _{T}}. \end{aligned}$$

If a binary mixture is heated from below ($Ra_T>0$), the required temperature difference to induce convection decreases as $\psi$ increases, while a binary mixture heated from above ($Ra_T<0$) can undergo convection only for $\psi<\psi _{lim}<0$ and the absolute value of the temperature difference to induce convection decreases at increasingly negative $\psi$ [2–4]. Here, $\psi _{lim}=-Pr/(Pr+Sc)\approx -Pr/Sc$, where the Prandtl number is $Pr=\nu /\kappa$, the Schmidt number $Sc=\nu /D$.

The situation becomes even more complicated in the case of a ternary mixture, because the density profiles given by temperature and by the different fluid components need to be carefully evaluated in order to predict the stability of the mixture for very large times. The differences in the time scales also play a major role in the behaviour of the fluid with respect to convective instabilities, as we will discuss later on.

The presence of convective instabilities in ground-based experiments calls for the need of performing experiments under reduced-gravity conditions [5]. This is one of the main reasons at the basis of the projects Diffusion Coefficient Measurements in ternary Mixtures (DCMIX) [6, 7] and Giant Fluctuations [8, 9]. Both projects aim at investigating ternary mixtures in microgravity. DCMIX was aimed at detecting the density profile in ternary mixtures under thermal stress by means of optical digital interferometry [6, 7], while Giant Fluctuations will investigate the transport properties of several complex mixtures by means of dynamic Shadowgraphy as a tool to investigate non-equilibrium fluctuations in ternary liquid mixtures stressed by a thermal gradient [8, 9]. In both experiments, a dilute polystyrene dissolved in an equimassic toluene-cyclohexane mixture was investigated as a model for ternary mixtures due to the large difference in the diffusion matrix eigenvalues, allowing the separation of the dynamical signals coming from the polystyrene from the one coming from the binary molecular solvent and, of course, from the even faster thermal component.

The special interest of studying this mixture under microgravity conditions relies on suppressing possible convective instabilities associated with the negative Soret coefficient of the binary solvent toluene–cyclohexane [10].

In the present paper, we report the shadowgraph observation of convective instabilities after the sudden imposition of a temperature gradient by heating the sample from above. The instability appears after a diffusive time related to the binary molecular solvent of toluene and cyclohexane and disappears after a larger diffusive time related to the mass diffusion of the polystyrene in the binary solvent. The mechanisms behind this behaviour will be discussed showing a non-trivial dependence on the parameters of the ternary mixture, both in terms of the intensity of the different mechanisms and on their evolution in time.

## Materials and methods

The investigated sample is a ternary liquid mixture consisting of polystyrene (M$\omega$ = 4730 g/mol, DIN-Poly(styrene) 4730 DA, PDI 1.03 by PSS-polymer), toluene (Sigma–Aldrich, 24, 451-1, 99.8%) and cyclo-hexane (Sigma-Aldrich, 179191, 99%) with mass fractions of 2/49/49$\%$ w/w, so that $\omega _1$=0.02 and $\omega _2$=0.49. The relevant fluid properties are reported in Table 1. In the case of a mixture of three components, the mass diffusion coefficient actually reads as a $2\times 2$ matrix, but in the following we will refer only to its eigenvalues and call them simply $D_1$ and $D_2$.

Following the results from [11], close to infinite dilution of polystyrene, $D_2$ matches the Fick diffusion of the binary mixture toluene-cyclohexane and $D_1$ the tracer diffusivity of polystyrene [12, 13]. The latter value has been determined by the Stokes–Einstein relation using the hydrodynamic radius of polystyrene derived from diffusivity data of polystyrene in toluene [14, 15] and density data from [10]. The corresponding Soret coefficient $S_{T1}$ is that of an infinitely small amount of polystyrene dissolved in pure toluene [14, 15]. Even if this value represents only an estimation, an error of 50% on $S_{T1}$ has no consequences on the results discussed in the following.

In analogy to binary mixtures, we also define two separation ratios: $\psi _1=\omega _1(1-\omega _1)S_{T_1}{\beta _{\omega _1}}/{\beta _{T}}$ and $\psi _2=\omega _2(1-\omega _2)S_{T_2}{\beta _{\omega _2}}/{\beta _{T}}$. Following the theoretical development described in reference [16], we define also a global separation ratio $\varPsi =\psi _1+\psi _2$ aimed at describing the overall separation ratio of the ternary mixture induced by the Soret effect.

Our apparatus involves two main parts: a thermodiffusion cell allowing precise differential temperature control over a precise layer of the liquid mixture and the optical setup aimed at acquiring Shadowgraph images of the density variations within the fluid. The light beam passes through the sample in the vertical direction, i.e. parallel to the imposed temperature gradient. The optical access is obtained by sandwiching the sample between two sapphire windows of size 40x40x8 mm$^3$. The thermodiffusion cell has been widely utilised in our laboratory for investigating both non-equilibrium fluctuations and convective instabilities [17–26]. The sample is confined horizontally by a Viton O-ring of about 20 mm diameter and vertically by the two sapphire windows that are kept apart by three Delrin spacers of $(2.00\pm 0.01)$ mm. Each sapphire window is temperature controlled by a square Peltier element with an inner hole of diameter 13 mm controlled by a thermo-electric-controller device. A thermistor placed close to each sapphire window acts as temperature detector. The RMS stability of the two temperatures applied to the sapphire windows is of the order of 1 mK over 24 hours. The uniformity of the temperature gradient across the observation window is better than 5$\%$ as previously checked by finite elements numerical simulations. The cell assembly can be tilted, and its horizontal alignment is carefully checked by means of a bubble level. The entire system is mounted on a kinematic table including pneumatic isolation from ground vibrations.

**Table 1:** Thermophysical properties of the mixture polystyrene/toluene/cyclohexane at 2/49/49$\%$ w/w. Data are taken from Refs. [10, 14–16]

Parameter	Value
$\rho$	$({811}\pm 5)\times 10^{-3}$ $\mathrm { g/cm^3}$
$\nu$	$(7.4\pm 0.1)\times 10^{-3}$ $\mathrm {cm^2/s}$
$\kappa$	$(9.0\pm 1)\times 10^{-4}$ $\mathrm {cm^2/s}$
$D_2$	$(1.9\pm 0.3)\times 10^{-5}$ $\mathrm {cm^2/s}$
$D_1$	$({2.4}\pm 0.2)\times 10^{-6}$ $\mathrm {cm^2/s}$
$\beta _{T}$	$(1.0\pm 0.3)\times 10^{-3}$ $\mathrm {1/K}$
$\beta _{\omega _2}$	$(0.11\pm 0.05)$
$\beta _{\omega _1}$	$(0.30\pm 0.08)$
$$ S_{T_2}$$	$$-(2.1\pm 0.2)\times 10^{-3}$$ $$\mathrm {1/K}$$
$$ S_{T_1}$$	$$({4.6}\pm 0.5)\times 10^{-2}$$ $$\mathrm {1/K}$$
$\psi _{2}$	$-(0.06\pm 0.01)$
$\psi _{1}$	$({0.27}\pm 0.02)$

**Fig. 1:**
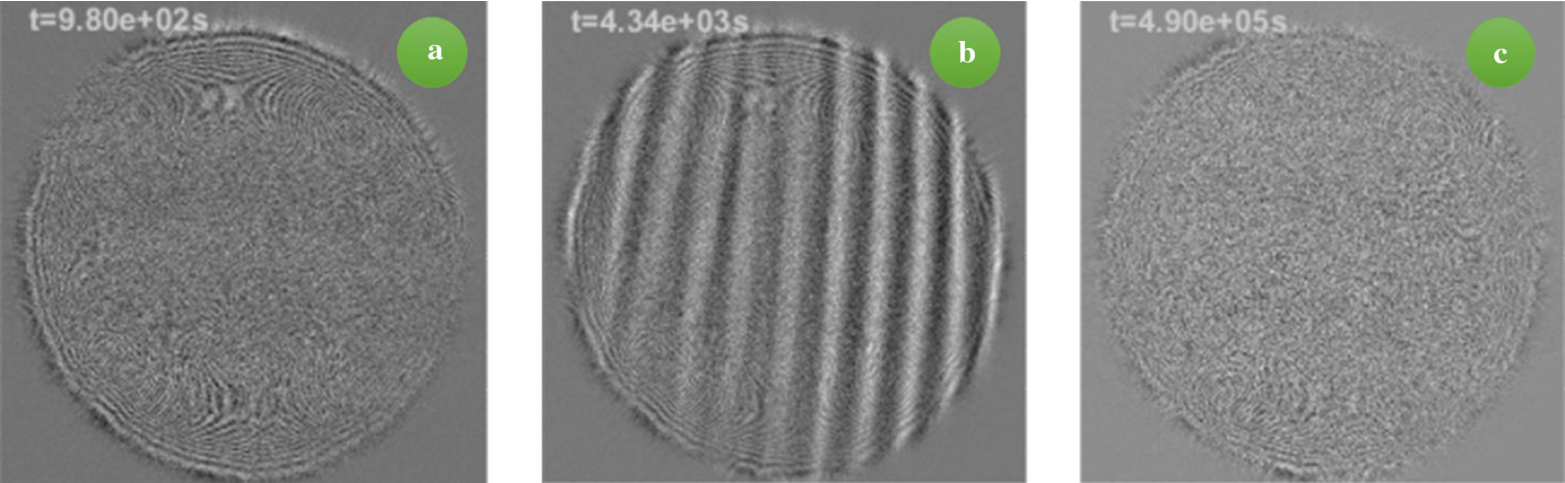
Shadowgraph difference image of the convective pattern for $L=2$ mm and $\varDelta T=20$ K. **a** During the formation of the destabilizing concentration profile of the toluene/cyclohexane binary solvent; the side corresponding to 15 mm, **b** during the roll-type convective instability and **c** after the formation of the stabilizing concentration profile of the polystyrene in the binary solvent. A movie is available as Supplementary Material

The shadowgraph is a convenient optical method thanks to the simplicity of the optical setup and the absence of need for calibration [27–30]. It allows to visualize and measure changes of the index of refraction $\delta n$, which are related to density variations in the fluid as originated by temperature and concentration variations: $\delta \rho = \delta T\cdot \partial \rho /\partial T+\delta \omega \cdot \partial \rho /\partial \omega$. Here, we assume that pressure variations are negligible in the system, otherwise an additional term would be needed to describe the total density variations. The optical layout is made by a super-luminous diode (Superlum, SLD-MS-261-MP2-SM) $\lambda =(675\pm 13)$ nm and a maximum intensity of about 5 mW as a light source coupled to a monomode optical fibre. A collimating lens (an achromatic doublet with a focal length of 250 mm) is positioned at a focal distance from the optical fibre output. This makes the beam plane parallel with a width at $1/e^2$ of about 2 cm of diameter so that it can illuminate the sample with a roughly homogeneous intensity. The beam then crosses the sample in the vertical direction and is captured by a Charged Coupled Device (CCD) detector (AVT, PIKE-F421B) with a resolution of $2048\times 2048$ pixels, each pixel having a size of $7.4\times 7.4$ $\mathrm {\mu m^2}$ and a resolution of 14-bit. No additional lens is present in the collection part of the optical setup.

Starting from the application of the temperature gradient images are acquired at variable frequency from 1 Hz down to 0.01 Hz, in order to follow the evolution of the image contrast over a wide range of time up to about 600,000 s, corresponding to about one week. The image contrast is defined as $C(t)=\langle [i(\varvec{x},t)-i(\varvec{x},t_0)]^2\rangle _{\varvec{x}}$ where $\langle ...\rangle _{\varvec{x}}$ represents the average over the pixels of an image, $i(\varvec{x},t)=I(\varvec{x},t)/\langle I(\varvec{x},t) \rangle _{\varvec{x}}$ is an image normalized by its spatial average , and $i(\varvec{x},t_0)$ is a normalized background image at time $t_0$ before applying the temperature gradient.

## Experimental results

The measurements reported here have been performed by imposing a temperature difference of $\varDelta T=20$ $\mathrm {K}$ to the sample so that the temperature profile generated in the fluid mixture is gravitationally stable, i.e. the temperature at the fluid top is higher than that at its bottom. Therefore, the thermal density difference is negative $\varDelta \rho _T=\rho _T|_{z=L}-\rho _T|_{z=0}<0$. The linear temperature profile is rapidly established within the fluid layer, in a time of the order of $\tau _{th}=L^2/\kappa$, where *L* is the vertical thickness of the sample and $\kappa$ is the thermal diffusivity of the mixture. In the present case, $\kappa =9\times 10^{-4}$ $\mathrm {cm^2/s}$ and $L=2$ mm, so that the resulting thermal time constant is $\tau _{th}\approx 45$ s. We can therefore consider that the thermal profile is well established after about 4 minutes. Eventually, the concentration profile builds-up induced by thermodiffusion and the evolution of the system is shown in Fig. 1.

The concentration profile related to the molecular binary solvent toluene and cyclohexane is considerably slower to establish due to the large Lewis number of the liquid binary solvent, here $Le_2=\kappa / D_2 \approx 47$, meaning that the concentration time constant will be about 47 times larger than the thermal one. We can anyway compute the solutal time constant as $\tau _{2}=L^2/D_2$ using the mass diffusion coefficient of the binary solvent $D_2=1.9\times 10^{-5}\,\mathrm {cm^2/s}$ and obtain $\tau _{2}\approx 2,100$ s. Therefore, to get a well-established concentration gradient one should wait about 3 hours.

Eventually, the concentration profile resulting from the Soret separation of the polystyrene is formed at an even larger timescale given by the mass diffusion coefficient of the polystyrene in the binary solvent, $\tau _{1}=L^2/D_1$ and using the mass diffusion coefficient of the investigated sample $D_1={2.4}\times 10^{-6} \mathrm {cm^2/s}$ one obtains $\tau _{1}\approx {17,000}$ s; therefore, to get a well-established concentration gradient one should wait about 24 hours.

The polystyrene solutal diffusive time can also be computed by multiplying the thermal one by the relevant Lewis number $Le_1=\kappa / D_1\approx {380}$ or by multiplying the binary solvent solutal diffusive time by the diffusion eigenvalue ratio $Dr=D_2/D_1\approx {8}$ [13, 31].

**Fig. 2:**
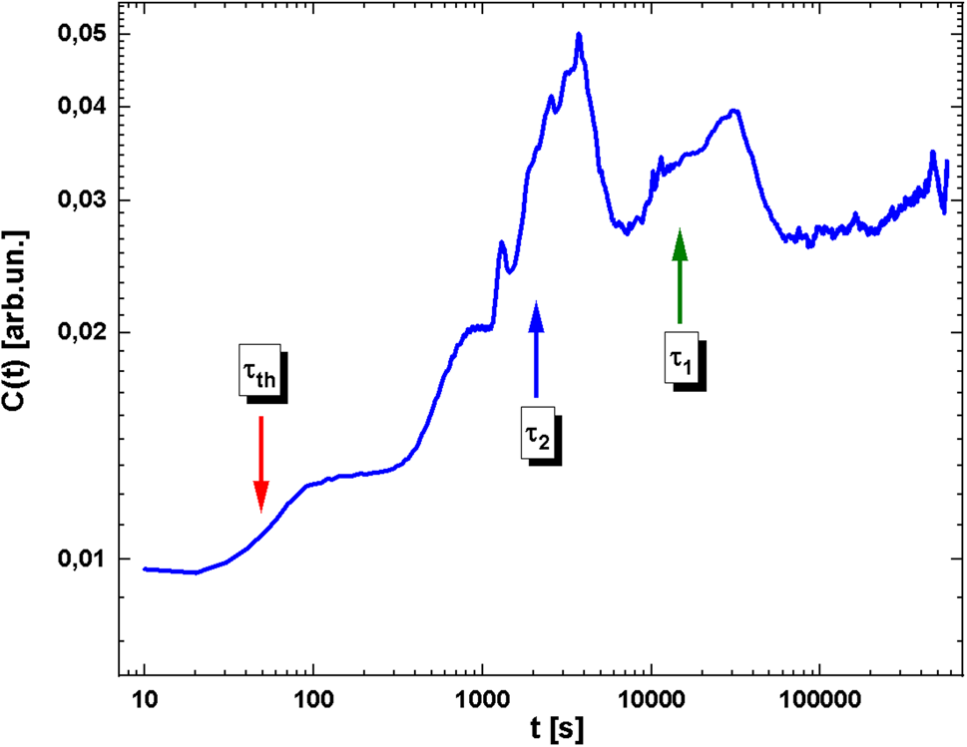
Optical contrast of shadowgraph images *C*(*t*) as a function of time. The vertical arrows indicate the three different time constants for temperature, and concentration

A few seconds after imposing the temperature difference to the external sides of the two sapphire windows, a linear temperature profile develops within the liquid and temperature fluctuations are amplified by the presence of the temperature gradient increasing their intensity. This is revealed by the small increase in the optical contrast visible in Fig. 2 around $\tau _{th}$ and is related to the increasing intensity of temperature NE fluctuations due to the increasing temperature gradient developing within the fluid layer. The density difference provided by the temperature gradient is given by:

4 $$\begin{aligned} \varDelta \rho =\varDelta \rho _T=-\rho \beta _T \varDelta T . \end{aligned}$$

An estimation of the total density difference in our experimental conditions for $\beta _T=1\times 10^{-3}$ $\mathrm {1/K}$ and $\varDelta T = 20$ $\mathrm {K}$ provides $\varDelta \rho =-{16.2}\times 10^{-3}$ $\mathrm {g/cm^{3}}$. We compute the associated thermal Rayleigh number by means of Eq. 1; $Ra_T\approx$ $-23,500$. It is trivial to note that the resulting Rayleigh number stands for a stable situation, i.e. $Ra_T<Ra_{T_c}=1707.8$, since the sample is heated from above and $\beta _T$ is positive, as for most liquids at ordinary thermodynamic conditions.

On a larger time scale a concentration gradient, generated by the Soret effect on the binary solvent of toluene and cyclohexane, builds up. This is again visible from the optical contrast graph in Fig. 2, where a second increase is visible around $\tau _2$. Interestingly, this increase in the optical contrast initially reaches a plateau and eventually starts increasing again, after about 2, 000 s when linear convective rolls are visible in the images (see Fig. 1b). The density difference across the sample is now due to both the temperature and the concentration profiles, where for concentration, we refer to the concentration of the binary solvent of toluene and cyclohexane. The overall density difference is thus:

5 $$\begin{aligned} \varDelta \rho =\varDelta \rho _T+\varDelta \rho _{\omega _2}=-\rho \beta _T \varDelta T +\rho \beta _{\omega _2} \varDelta \omega _2 . \end{aligned}$$

The density difference relative to the concentration of the binary solvent is then $\varDelta \rho _{\omega _2}=\rho \varDelta \omega _2 \beta _{\omega _2}$, where the concentration gradient originated by the Soret effect can be written as:

6 $$\begin{aligned} \varDelta \omega _2=-\omega _2(1-\omega _2)S_{T_2}\varDelta T, \end{aligned}$$

where $S_{T_2}=-2.1\times 10^{-3}$ $\mathrm {K^{-1}}$ is the Soret coefficient of the binary solvent of toluene and cyclohexane and $\omega _2=0.49$ is the concentration of component 2, i.e. the toluene. The calculated concentration difference induced by the Soret effect on the binary solvent is thus $\varDelta \omega _2=0.01$. The density difference is then $\varDelta \rho _{\omega _2}=+1.0\times 10^{-3}$ $\mathrm {g/cm^{3}}$. Thus, the total density gradient is $\varDelta \rho =\varDelta \rho _T+\varDelta \rho _{\omega _2}=-{15.2}\times 10^{-3}$ $\mathrm {g/cm^{3}}$. In this case, the total density gradient remains negative and one might think that the fluid layer is still stable against convection. We then calculate the solutal Rayleigh number relative to the binary solvent mixture through Eq. 2; $Ra_{\omega _2}\approx$ $+64,100$. This value is definitely larger than the critical solutal Rayleigh number $Ra_{\omega _c}=720$, so that linear stability analysis suggests that the system is unstable and convective rolls should take place in the system. That is what we have observed in our experiments for intermediate times between 1,000 and 10,000 s. This observation is in agreement with the results obtained for the binary mixture of water and isopropanol (90/10 w/w) in reference [32].

The patterns visible in Fig. 1b correspond to linear convective rolls, as one should expect for solutal convection induced by thermodiffusion in a mixture with negative Soret coefficient [33]. For intermediate times, in fact, the mixture behaves quite similar to the toluene–cyclohexane binary mixture [10].

This shows that the transient behaviour of the system is driven only by the density gradient relative to the solutal component $\varDelta \rho _{\omega _2}$. Another way of considering the problem can be that of considering non-equilibrium fluctuations (NEFs). Here, concentration NEFs have time constants that are much larger than temperature NEFs and are therefore more persistent. The effect of gravity at the peak wavelength promotes the amplification of concentration fluctuations and the establishment of convection even in this specific case, where the overall density profile is stable.

**Fig. 3:**
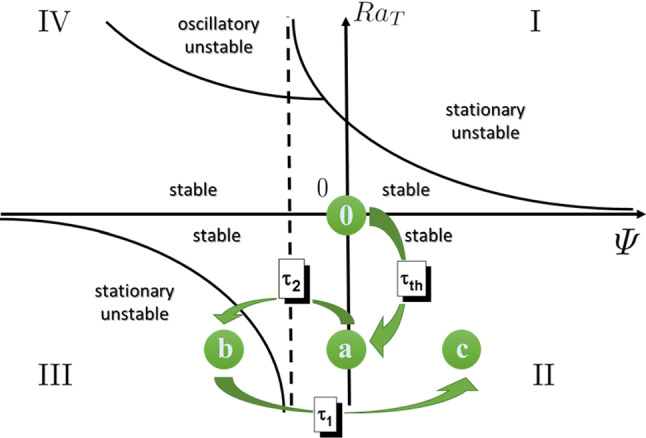
Time evolution of the system state (not in scale) in the stability diagram adapted from reference [2]

An additional consideration is that the separation ratio for the toluene and cyclohexane mixture is $\psi _2=-0.06$. According to the stability diagram shown in Fig. 3 (adapted from Ref. [2]), quadrant III, for $\psi _2$ smaller than the threshold $\psi _{lim}=-0.02$ stationary convection should be present for long times.

The situation changes again when the concentration gradient promoted by the Soret effect of the quasi-binary mixture consisting of polystyrene diluted in the binary solvent is fully developed. This concentration profile is still supposed to be linear with a good approximation, given the rule of thumb stated by Köhler and coworkers [34], stating that the linearity is assured if the temperature difference applied to the mixture is smaller than the inverse of the Soret coefficient. In our experiments, the Soret coefficient relative to the polystyrene is $S_{T1}={4.6}\times 10^{-2}\mathrm {1/K}$, so that its inverse is about 22 $\mathrm {K}$, while the applied temperature difference is 20 $\mathrm {K}$, so that the condition $\varDelta T< 1/S_{T1}$ is roughly met.

This happens well after $\tau _1{\approx 17,000}$ s, i.e. the diffusive time of polystyrene in the binary solvent. At very long times the density difference within the sample can be written as the sum of three components:

7 $$\begin{aligned} \varDelta \rho= & {} \varDelta \rho _T+\varDelta \rho _{\omega _2}+\varDelta \rho _{\omega _1}\nonumber \\= & {} -\rho \varDelta T \beta _T+\rho \varDelta \omega _2 \beta _{\omega _2}+\rho \varDelta \omega _1 \beta _{\omega _1}. \end{aligned}$$

The density difference relative to the concentration of the polystyrene is now $\varDelta \rho _{\omega _1}=\rho \varDelta \omega _1 \beta _{\omega _1}$, and the concentration difference due to the Soret effect is:

8 $$\begin{aligned} \varDelta \omega _1=-\omega _1(1-\omega _1)S_{T_1}\varDelta T. \end{aligned}$$

Given the value of $S_{T_1}$ cited above and $\omega _1=0.02$, one can calculate the concentration difference induced by the Soret effect: $\varDelta \omega _1=-{0.018}$. The density difference is then $\varDelta \rho _{\omega _1}=-{4.4}\times 10^{-3}$ $\mathrm {g/cm^{3}}$. Thus, the total density gradient is $\varDelta \rho =-{19.6}\times 10^{-3}$ $\mathrm {g/cm^{3}}$. As expected, the total density difference is again negative and the overall density profile is stabilizing. Finally, we calculate the solutal Rayleigh number relative to the polystyrene dissolved in the binary solvent through Eq. 2; $Ra_{\omega _1}\approx$ $-{2,430,000}$. The latter indicates again a stable condition due to the negative sign. It is therefore straightforward to expect a stable configuration without any convective instability for times much larger than the mass diffusive time of the polystyrene over the cell thickness.

The separation ratio for a polystyrene solution is now $\psi _1={0.27}$, confirming the stability of the system. Also, the overall separation ratio $\varPsi =\psi _1+\psi _2={0.21}$ is again confirming the stability of the system, see quadrant II of the stability diagram in Fig. 3.

## Discussion and conclusions

The results obtained above show that the density profile obtained after applying a temperature gradient to the sample is always ‘stable’ in the sense that the total density is always larger at the bottom of the fluid. This is compatible with the idea that it is not the macroscopic total density profile that leads the convective instability, but a combination of the boundary conditions with local density mismatch and the dynamical behaviour, witnessed by the separation ratio. Another way to express this concept is that, given the different time scales, fluctuations of temperature are affected by gravity independently from fluctuations of concentration. That is why, even if the overall density difference is negative, concentration fluctuations of the binary solvent are amplified by gravity and induce the transient convective motion that is visible in the reported experiment for intermediate times, while temperature fluctuations relax fast and the portion of fluid where the fluctuation occurred becomes rapidly neutrally buoyant with respect to thermal expansion [35–37].

The effect of gravity on fluctuations in temperature and on fluctuations in concentration of the polymer is that of decreasing their amplitude, even if in different way, given the fact that boundary conditions are not the same for temperature and concentration. The different time scales play, at the end, a major role on the stability of the fluid mixture. In our case the final, total separation ratio $\varPsi$ is positive, representing a stable steady-state. It would be interesting to find a sample for which the total $\varPsi$ is negative and smaller than the limit value for stationary unstable condition, but the $\psi _1$ is positive and check what is the stability of the system.

Moreover, in this paper we report results for a single (thermal) Rayleigh number, but it would be interesting to investigate more values. Different values of the Rayleigh number can be achieved either by changing the temperature gradient or the cell thickness *L* (with a dependence to $L^3$). In the former case, the linearity of the concentration gradient would be affected [34], especially for temperature differences larger than $S_T^{-1}$, while in the latter it will not. This would allow to carefully check the impact of the gradient linearity, but this study is outside the scope of the present paper.

In this work, we report experiments performed by imposing a temperature difference to a thin layer of fluid heating from above. The fluid consists of a mixture of three components, namely polystyrene, toluene and cyclohexane. This mixture is known to have one positive and one negative Soret coefficient, so that the stability of the mixture is not trivial at all. In our case, i.e. applying a temperature difference of 20 K over a layer of 2 mm, the system is stable for short times of the order of tens of seconds, then unstable for intermediate times of the order of hundreds of seconds and, at the end, stable again for large times of the order of thousands of seconds. This observation confirms the fact that the Soret coefficient of the binary solvent toluene–cyclohexane is negative, so that heating from above ends up with concentration-driven convection and linear convective rolls. It also confirms that the Soret coefficient of the quasi-binary mixture of polystyrene diluted in the binary solvent toluene–cyclohexane is positive. The relative amplitude of the generated density gradients is such that the system becomes stable again. The final stability of the system is compatible with the observation and analysis of temperature and concentration non-equilibrium fluctuations and, thus, the retrieval of transport properties of the fluid, like thermal and mass diffusion coefficient, as well as the two Soret coefficient even in normal gravity conditions. This will be the subject of a further paper. The information obtained on ground in this work will be of great interest in comparison with the data obtained in microgravity conditions.

## Supplementary Information

Below is the link to the electronic supplementary material.

Supplementary file 1 (mp4 2786 KB)
